# Supplementary material for: Imaging Peripheral Nerves In Vivo with CT Neurogram Using Novel 2,4,6-Tri-Iodinated Lidocaine Contrast Agent
Source: Bioengineering (Basel). 2025 Apr 16;12(4):422. doi: 10.3390/bioengineering12040422 (PMC12024922; doi:10.3390/bioengineering12040422)

SUPPLEMENTAL INFORMATION

Imaging the peripheral nerves in vivo with CT neurogram using novel tri-iodinated lidocaine contrast agent

Rui Tang, PhD[a*], Ron Perez[b*], David M. Brogan, MD[b], Mikhail Y. Berezin, PhD[a#], James E. McCarthy, MD[c#]

[a] Washington University School of Medicine, Department of Radiology, St. Louis, MO 63110, USA

[b] Washington University School of Medicine, Department of Orthopedic Surgery, St. Louis, MO 63110, USA

[c] MacHouse Designs, LLC

* The authors contributed equally

# corresponding authors: berezinm@wustl.edu, mccarthymd@gmail.com

SRI Biosciences™
A DIVISION OF SRI INTERNATIONAL

Chemical Data Sheet

**Sample ID:** 17069-LL-86<br><br>**To:**<br>**Dr. James McCarthy**	**Contractor:** SRI International<br>333 Ravenswood Avenue<br>Menlo Park, CA 94025<br><br>**Principal Investigator**: Dr. Leyi Gong<br>**Project No**: 101630.000.0000.00.0000
**Chemical Name:** 2-(diethylamino)-N-(2,4,6-triiodophenyl)acetamide	

**Structure**	**Molecular Formula:** C12H15I3N2O
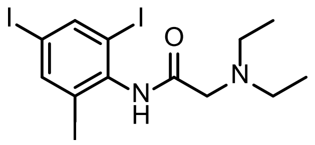	**Molecular Weight:** 583.98
	**Preparation Date:** 03/25/2024
	**Notebook Number:** 17069-LL-86
	**Amount:** 340 mg
	**Synthetic Chemist:** Lisa Liu, M.S.
	**Analytical Chemist:** Lori Olson, M.S.
	**Reviewer:** Leyi Gong, Ph.D.
	**Measured purity:** 98.0%

Sample description: Off-white solid.

^{1}H NMR: (300 MHz, MeOD) δ8.24 (s, 2H), 3.24 (s, 2H), 2.75 (q, 4H), 1.16 (t, 6H)

LCMS: Thermo-Fisher, Agilent Pursuit C18 2x50 mm; flow rate 0.5 mL/min, UV: 254 nm, solvent 5-95% MeCN (0.1% formic acid) in H_2O (0.1% formic acid) over 10 min, Rf = 3.63 min; **MS:** (ESI positive ion mode) m/z 584.61 (M+1).

1H NMR

17069-86-fp

Sample Name	17069-86-fp	Pulse sequence	PROTON	Temperature	23	Study owner	walkup
Date collected	2024-03-25	Solvent	cd3od	Spectrometer	mercury300-mercury300	Operator	walkup

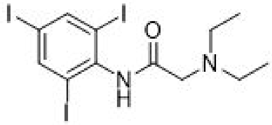

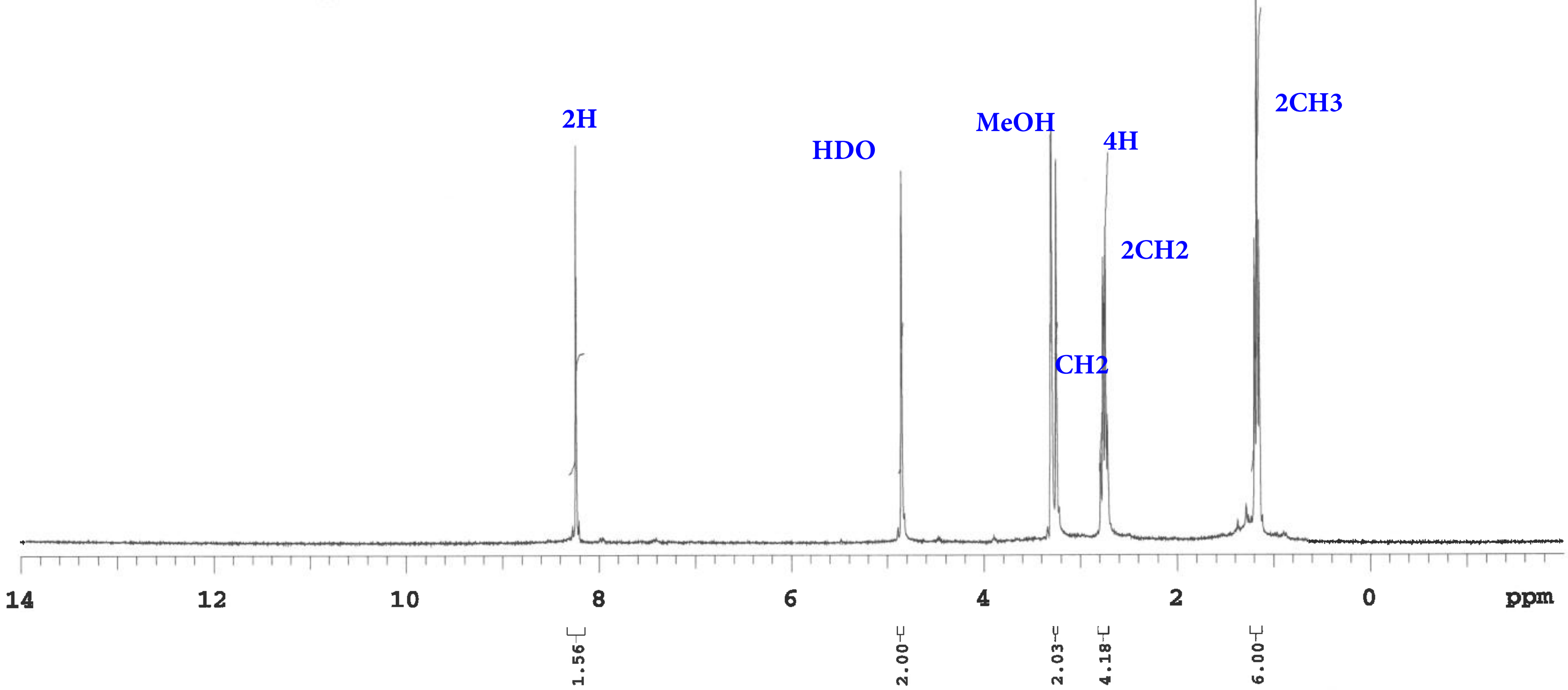

Data file /home/walkup/vnmrsys/data/17069-86-fp_20240325_01/PROTON_01

Plot date 2024-03-25

D:\Lisa\~17069\17069-86-pk2 **LCMS** 03/21/24 14:29:04 **Triiodo-lidocaine**

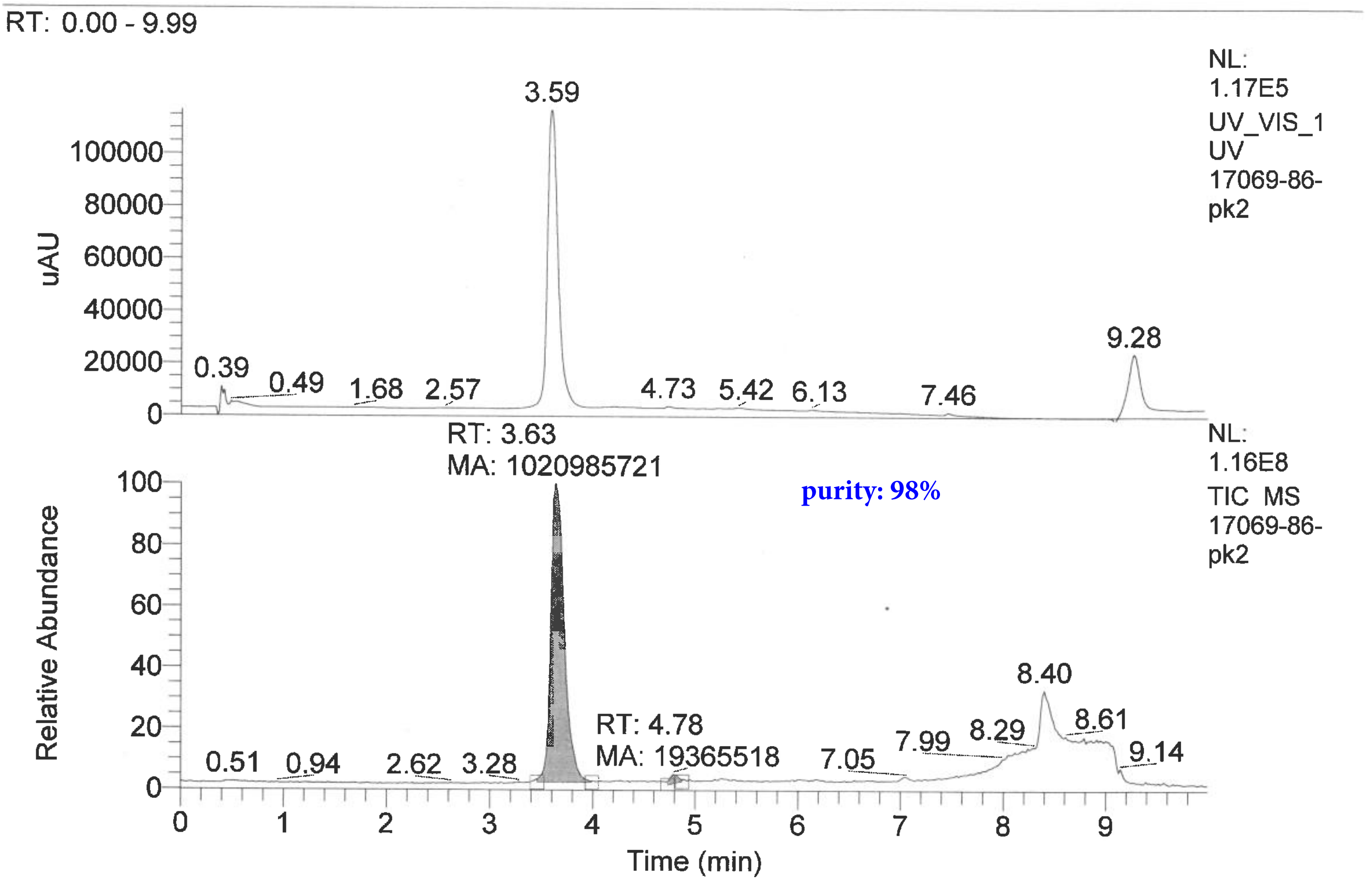

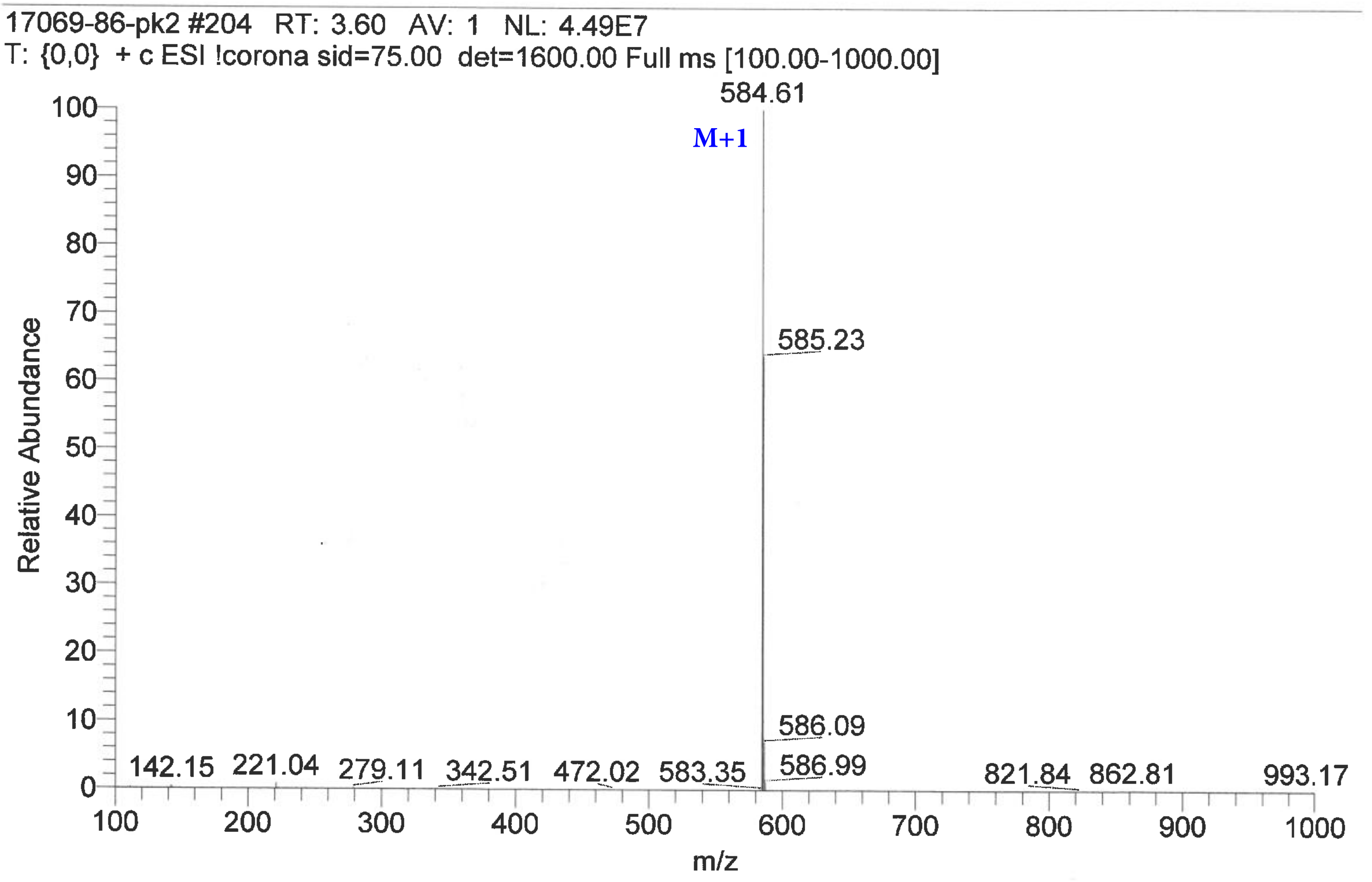

Supplement: Supplementary file 1 [file bioengineering-12-00422-s001.zip › bioengineering-3554701-supplementary (1).pdf]
